# Lessons from the National institutes of health innovation corps program: defining barriers to developing and commercializing novel solutions for persons with opioid use disorder

**DOI:** 10.1186/s13722-025-00554-1

**Published:** 2025-03-12

**Authors:** Matthew P. Heshmatipour, Tyler M. Duvernay, Desislava Z. Hite, Eboo Versi, Michael P. Hite, David F. Reeser, Victor Prikhodko, Ariana M. Nelson, Bina Julian, Milton L. Greenberg

**Affiliations:** 1 The Substance Use Disorder Solutions Network, Wilmington, United States; 2 School of Medicine, University of California, Irvine, United States; 3 Department of Anesthesiology and Perioperative Care, University of California, Irvine, United States; 4 Department of Physiology and Biophysics, School of Medicine, University of California, Medical Sciences D350, Irvine, 92697 CA United States; 5 Department of Obstetrics, Gynecology and Reproductive Sciences, Rutgers University, New Brunswick, United States

**Keywords:** Opioid use disorder (OUD), Stigma, Clinicians, Medical devices, Therapeutics commercialization

## Abstract

**Background:**

Translating innovative research advancements into commercially viable medical interventions presents well-known challenges. However, there is limited understanding of how specific patient, clinical, social, and legal complexities have further complicated and delayed the development of new and effective interventions for Opioid Use Disorder (OUD). We present the following case studies to provide introductory clinical, social, and business insights for researchers, medical professionals, and entrepreneurs who are considering or are currently developing medical.

**Methods:**

Four small business recipients of National Institute on Drug Abuse (NIDA) small business grant funding collected a total of 416 customer discovery interviews during the 2021 National Institutes of Health (NIH) Innovation-Corps (I-Corps) program. Each business received funding to advance an OUD-specific innovation: therapeutics (2 companies), medical device (1 company), and Software as a Medical Device (SaMD) (1 company). Interview participants included stakeholders from a variety of disciplines of Substance Use Disorders (SUD) healthcare including clinicians, first responders, policymakers, relevant manufacturers, business partners, advocacy groups, regulatory agencies, and insurance companies.

**Results:**

Agnostic to the type of product (therapeutic, device, or SaMD), several shared barriers were identified: (1) There is a lack of standardization across medical providers for managing patients with OUD, resulting in diverse implementation practices due to a fragmented healthcare policy; (2) Underlying Social Determinants of Health (SDOH) present unique challenges to medical care and contribute to poor outcomes in OUD; (3) Stigma thwarts adoption, implementation, and the development of innovative solutions; (4) Constantly evolving public health trends and legal policies impact development and access to OUD interventions.

**Conclusion:**

It is critical for innovators to have early interactions with the full range of OUD stakeholders to identify and quantify true unmet needs and to properly position development programs for commercial success. The NIH I-Corps program provides a framework to educate researchers to support their product design and development plans to increase the probability of a commercially successful outcome to address the ongoing opioid epidemic.

## Background

The opioid epidemic remains an overwhelming threat to public health across the United States. From 2017 to 2021 opioid-related deaths in the United States increased to 69%, with over 80,000 deaths reported in 2021 [1, 2]. The receding of the COVID-19 pandemic and preventative measures like restrictive prescribing practices [3, 4] and nationwide educational campaigns [5] helped slow the rates of individuals developing Opioid Use Disorder (OUD). However, we are largely failing to improve outcomes for persons with OUD, and there is insufficient support and healthcare for patients who are actively using illicit substances and a concomitant increase in morbidity or mortality from drug overdose (OD). Despite the proven efficacy of Medications for OUD (MOUD), also known as Medication-Assisted Treatment (MAT), a staggering 88% of the three million Americans with OUD do not receive MOUD to support a path to recovery [6].

Several barriers to treatment access [7–10] combined with distinct challenges [1, 11] from each new wave of the opioid crisis create a steep and rapidly shifting learning curve for clinicians and caregivers as they try to meet the pharmacological and psychological needs of patients. A fragmented understanding of patient and societal needs compounds this problem, reflected in well-intentioned clinician curricula, medical interventions, and harm reduction programs that may quickly require adjusting or become outdated from the unique challenges of each wave of the epidemic. Current trends of polysubstance ODs [12, 13], an alarming increase in vulnerable populations, such as minors, ethnic minorities, and patients of lower socioeconomic status accessing synthetic and prescription opioids [14], and the impact of fentanyl analogs on initiation of MOUD [15] is not sufficiently addressed by existing tools and knowledge. There is an urgent need to improve and integrate our understanding of social, economic, medical, and commercial factors to improve outcomes for OUD sufferers. Barriers to market entry are unique for each novel OUD intervention and require a framework for researching and mitigating challenges that may prevent successful commercialization.

### National institutes of health innovation corps (NIH I-Corps)

The Helping to End Addiction Long-term (HEAL) Initiative included small business programs, including NIH-funded Small Business Innovation Research (SBIR) and Small Business Technology Transfer (STTR) grant programs [16]. HEAL-funded SBIR/STTR projects act to encourage private-sector commercialization of technologies and products to prevent, diagnose, and treat OUD and OD, as well as to safely enhance pain management.

The NIH Innovation Corps, or I-Corps, program [17, 18] is an entrepreneurship training program offered to SBIR/STTR awardees. The NIH I-Corps program was adapted from similar federal programs [17] to provide entrepreneurial training in the biotechnology, therapeutics, devices, diagnostics, and digital health sectors. Participants are provided a framework for customer discovery and research commercialization [18] and are forced “out of the lab” and into conversations with relevant stakeholders including clinicians, payors, and investors. The goal of the NIH I-Corps program is to enhance the commercialization potential of these NIH-funded technologies to ensure an early understanding of unmet medical and commercial needs thus enabling innovators to develop commercialization plans at an early stage to increase the probability of private financing.

In 2021, NIDA took a large role in the NIH I-Corps program, enrolling eight OUD-focused companies into the April 2021 cohort. Four participating companies have collaborated to author this publication, representing an aggregate of 416 interviews with various stakeholders within the OUD community. Each case study details the I-Corps experience of a small business focused on an innovative tool to address the needs of OUD in the following areas: development of novel therapeutic, medical device, and digital health innovations.

## Methods

### NIH SBIR I-Corps program design

NIDA grant awardees were encouraged to apply to a supplemental funding mechanism titled, “Innovation Corps (I-Corps™) at NIH Program for NIH and CDC Translational Research (Admin Supp Clinical Trial Not Allowed)” through the PA-19-029 funding opportunity. The selection process consisted of a supplemental grant application and a virtual team interview with representatives of NIH contractor VentureWell, Inc. and the NIH. Selected companies were required to form three-person teams comprised of a C-Level Corporate Officer, Principal Investigator, and an Industry Expert. The I-Corps course was run by lecturers and business coaches from VentureWell in a fully remote format due to restrictions imposed at the time in response to the COVID-19 pandemic.

Each week I-Corps teams completed mandatory reading assignments [19, 20], pre-recorded business lecture sets (Launchpad Portal), course instructor office hours, customer discovery interviews, and delivered an oral presentation.

All members were required to participate in live classes, which were also attended by NIH observers. In these live classes, VentureWell instructors challenged the participants on specific topics (Table 1). Companies also presented their interview findings of the week by identifying the role of the interviewee in their respective ecosystem and sharing: *Here’s what we thought*,* Here’s what we did*,* and Here’s what we found*. After presenting, companies received feedback from instructors and other participants.

**Table 1:** A summarized outline of the classes that were administered during the NIH I-Corps program

Topic	Purpose
Value Propositions	Identify customer types and prioritize product features according to each type.
Revenue Streams, Channels, and Customer Relationships	Create a revenue strategy for the product by learning how customers will use your product, and what they will pay for and plan to grow your company’s offerings accordingly.
Key Activities, Partners, Costs, Resources	Build the company’s fundamental workflow and identify partnerships and resources needed for business growth.

### Stakeholder interviews

Groups were directed to collect their interviews according to the Business Model Canvas [17], starting with the Value Propositions and Customer Segments. Teams were required to conduct a minimum of 100 virtual or in-person interviews (10 interviews in the first week and 15 in every subsequent week). Interviews were conducted with clinicians, patients, and other OUD-related experts to provide a holistic understanding of the product market need and the barriers and facilitators encompassing the potential innovative tools.

During the last week of the program, the teams reflected on their previous seven weeks to develop a sense of what they have accomplished and how they can use their newly learned skills to progress their companies’ goals. Their presentations to the rest of the class consisted of the most critical and impactful lessons that were learned from the process. It entailed presenting the group’s business, and development and evolution of the business model canvas as the group progressed through the program.

## Results

### Case study 1 – therapeutics for the treatment of OUD

DMK Pharmaceuticals (DMK) was founded in 2016 in response to the rapidly intensifying U.S. opioid crisis. In March 2020, DMK received a Phase I HEAL SBIR award from NIDA to advance development of its lead small molecule DPI-125, a novel triple opioid agonist. The therapeutic goal of the DPI-125 program is to stabilize adult patients diagnosed with OUD by safely transitioning them from active opioid use into medical treatment mitigating the risks of opioid withdrawal and OD associated with initiating current Opioid Agonist Treatments (OAT) buprenorphine and methadone.

In April 2021, DMK’s Chief Executive Officer (CEO) and Chief Operating Officer (COO) participated in the I-Corps program to evaluate whether Emergency Departments (EDs) were the ideal clinical setting for the early adoption of DPI-125. EDs hold a frontline position in treating opioid ODs and are financially motivated to reduce high costs associated with poor outcomes and readmissions. The pre-discharge period presents an opportunity to guide patients who have recently recovered from a non-fatal OD toward MOUD; however, current OATs require moderate withdrawal or additional medical supervision. The DMK team proposed DPI-125 as a more patient-focused solution that would not require withdrawal and increase treatment efficiency with weekly transdermal patches and reduced clinic visits.

Pain point discovery interviews were conducted with 116 stakeholders, i.e. individuals who would administer, recommend, purchase, manufacture, sell, or influence the use and sale of an OAT or MOUD product. All but one interview was conducted via Zoom due to the limitations of the COVID-19 pandemic. Instead of a rigid list of pre-planned questions, the team prepared a bank of open-ended prompts to enable interviewees to elaborate on pain points related to treatment environment, pathways for patients to obtain care, MOUD selection and initiation, and general challenges to providing and maintaining patient care.

Of the 116 conversations, 49 were with healthcare providers treating substance use disorder, pain, emergencies, etc., while the remaining 67 were influential stakeholders in federal policy, United States Food and Drug Administration (FDA) regulation, insurance reimbursement, and product development. Key takeaways were selected on the concurrence of at least 5 distinct interviews and, unsurprisingly, reflect the complexities of the Opioid Crisis, capturing social, political, and technical challenges to implementing a treatment for OUD in EDs.

Major takeaways from the I-Corps interviews included the following:

1. Stigma creates barriers to MOUD initiation, including accounts of pharmacists refusing to fill buprenorphine prescriptions, ED staff showing cynicism towards connecting patients with external harm reduction services, and patients avoiding treatment due to family and employer concerns. Without significant social support, a patient recovering from a non-fatal OD is unlikely to be psychologically ready to initiate treatment.
2. X-waiver requirements, stipulated by the Drug Addiction Treatment Act of 2000, for prescribing buprenorphine were identified as a major obstacle to prescribing buprenorphine and since the writing of this paper have now been eliminated. However, the law remains a legal hurdle for commercialization due to its narrow listing of buprenorphine by name and its distribution restrictions for Schedule II MOUD. Only after a drug has achieved FDA approval does it receive DEA scheduling; the commercial viability of a novel Schedule II OAT is limited compared to a Schedule III OAT.
3. Most hospitals lack the necessary wraparound services for patients initiating MOUD post-discharge; Kaiser Permanente and Veterans Affairs were notable exceptions. The COVID-19 pandemic further disrupted existing and new ED programs supporting OUD recovery.
4. The pandemic led to increased SDOH awareness and adoption of harm reduction strategies, including telemedicine options and improved access to treatment through various state-level initiatives. This expanded access and treatment flexibility would commercially benefit a new Schedule III MOUD.
5. Pregnant women face unique barriers to care, as some states require automatic reporting to Child Protective Services for OUD medication use. This leads soon-to-be mothers to discontinue treatment postpartum or in the third trimester.
6. There is a paucity of clinical data and benchmarks for new OAT and MOUD, with current options (methadone and buprenorphine) originally developed and approved as analgesics. One interviewee with extensive FDA experience highlighted opioid-induced respiratory depression (i.e. the principal etiology of OD) as a key safety feature that lacks a standard clinical protocol and remains a barrier to innovation.

The I-Corps interviews revealed that gaps in post-discharge care and resource constraints within EDs would impede early adoption of DPI-125, which could compromise the commercial viability of a newly approved product. Instead, the company identified an optimal market entry strategy through addiction medicine specialists in inpatient and outpatient facilities. These specialists can provide consistent care and have the bandwidth to integrate new treatment protocols for a novel OAT. By identifying the most promising early adopters, DMK refined its product development strategy for initial market entry, with plans to expand to EDs at a subsequent stage.

### Case study 2 – therapeutics to mitigate opiate withdrawal symptoms

A second therapeutics company that wished to remain anonymous participated in the NIDA-funded I-Corps at NIH program to carefully define the appropriate product-market fit for their drug candidate. The therapeutic goal of their OUD program is to safely mitigate opioid withdrawal symptoms in adults voluntarily seeking treatment for OUD to facilitate immediate discontinuation of opioids. The CEO, COO, and Chief Scientific Officer (CSO) participated in the April 2021 I-Corps program to define the potential market need for the proposed therapeutic indication.

Currently approved drugs to treat opioid withdrawal can be divided into OATs, methadone and buprenorphine; and α2a adrenergic receptor agonists, lofexidine and clonidine [21]. In addition, a noninvasive, percutaneous electrical nerve field stimulator BRIDGE device is approved to mitigate withdrawal symptoms [22]. OATs have proven to be successful, with a 25 to 75% success rate of maintaining abstinence from illicit opioid use in patients with OUD [23, 24]. However, withdrawal will also be an issue if patients attempt to wean off their dose of OATs. Whereas OATs provide a mechanism for the replacement of illicit opioid use, non-opioid therapies and devices designed to work in combination with OATs improve outcomes by reducing withdrawal symptoms and permitting eventual tapering of OAT doses to lower levels with fewer adverse side effects (e.g. opioid-induced constipation).

Over the course of eight weeks, fifty clinicians from across the country who routinely treat patients with OUD were interviewed. They provided firsthand descriptions of the unmet clinical needs of patients suffering from OUD. Conclusions from these interviews largely fell into two broad categories: financial and social. While all the clinicians interviewed acknowledged the difficulties patients experience during withdrawal, the social factors that cause relapse and the added financial cost of seeing the same patient, multiple times a month, every month, in the same ED were the focus of the NIDA I-Corps discussions. Pivotal interviews were conducted with pharmacy and therapeutics committees from major academic medical centers. Pharmacy and therapeutics committees are responsible for bringing new medications into the institution, and the development and maintenance of the formulary. These clinicians stressed the importance of including pharmacoeconomic endpoints in future clinical studies to justify the adoption of novel therapeutics hoping to address withdrawal symptoms.

Major takeaways from the I-Corps research included the following:

1. There is extreme geographical diversity in the resources available for a community to standardize OUD treatment strategies. Certain medical centers readily prescribe α2a adrenergic receptor agonists for OUD patients experiencing withdrawal in an inpatient setting, while others rarely admit patients experiencing withdrawal and do not prescribe any treatments for the mitigation of withdrawal symptoms.
2. Since most OUD patients are underinsured, payors, including private insurance and Medicare, would likely not adequately reimburse for a new brand-name drug targeting persons suffering from OUD.
3. Major cost drivers that impact academic medical centers are the frequent, repeated admissions of the same OUD patient to the emergency department. Successful demonstration of mitigation of withdrawal symptoms that lead to a 10% reduction in the number of repeat ED admissions would be required for the adoption of a new brand-name drug targeting this patient population.
4. Raising investment around the OUD indication presents several challenges, including investor concern about future legal liabilities, uncertainties in developing a reimbursement strategy, the availability of generics, and the impact of comorbidities and SDOH in achieving primary clinical endpoints.

The research completed during the I-Corps program succeeded in defining the potential market need for a novel therapeutic to mitigate withdrawal symptoms in persons suffering from OUD. Strong clinical proof-of-concept data must be combined with convincing pharmacoeconomic data demonstrating reductions in repeat ED admissions. Together, these endpoints will make a strong case for both private insurance and Medicare to reimburse for this new therapeutic approach to reduce overall healthcare costs in both the short and long term. This combination of simultaneous therapeutic and financial benefits will position this program for third-party fundraising, from both a clinical and reimbursement perspective.

### Case study 3 – medical devices for opioid overdose

Ayuda Medical was founded with the vision of preventing medical emergencies at home from becoming fatal. Since 2020, Ayuda has been working to create a wearable (on-body) device to detect opioid OD and automatically deliver naloxone. In 2021, the CEO, Chief Medical Officer (CMO), and SUD Industry Expert participated in I-Corps to better understand the value for patients with OUD and to explore the adoptability of various options of response upon the detection of OD.

At onset, the company was prepared to create an automatic OD detector and naloxone autoinjector, because this would permit the fastest response and intervention. Other options for wrap-around protection included: an alarm, a speaker giving instructions to bystanders, a notification to patient-designated remote contacts, and an automatic call to 911.

During the program 103 people in the SUD community were interviewed at a rate of at least 15 interviews per week: patients, family members, counselors, clinicians, pharmacists. Some were in person (in three states: WA, CA, and WV), but many were virtual, which removed any travel restrictions and allowed greater flexibility in arranging interviews with different time zones.

A focus of the interview was the concerns and pain points of patients as they navigated the effects of opioids and OUD treatment in their community. Patients were also queried as to their personal risk of OD, previous experience of OD, and how it influenced current choices including whether to carry naloxone. A technique emphasized during I-Corps includes listening more than speaking, which was utilized in these interviews.

Major takeaways from the I-Corps interviews included the following:

1. Many OUD patients do want to notify someone if an OD is happening (as one member said: “We are not suicidal”), but many patients are also scared of the 911 responses and possible penalties that may occur following interactions with police and first responders.
2. Laws, consequences, and interventions vary greatly state to state and may change over time. Certain jurisdictions implement harm reduction methods, while others may threaten incarceration. These heterogeneous responses influence patient motivations and must be considered in development planning.
3. Interviewing stakeholders validated the need for an OD device in the home setting and helped us gather data showing the value of a new feature: Patient-Designated Contacts (DCs). DCs are people who have been pre-selected to be notified in case an OD is happening and will be pre-educated on ways they can respond if they were to receive an OD notification.

To conclude, I-Corps saved this company time and money by helping researchers understand features that would hinder adoption like automatic call to 911 and autoinjection of Naloxone, and those that will improve adoptions like patient chosen contacts. Another critical lesson learned was that Minimum Viable Product (MVP) design must address patient needs and concerns, and MVP design benefits from stakeholder input. For Ayuda Medical, this resulted in pivoting from a diagnostic device-drug autoinjector to a diagnostic device with Patient-Designated Contacts.

### Case study 4 – software as a medical device (SaMD)

OpiAID was founded in 2018 in Wilmington, North Carolina, where an estimated 11.6% of the city’s working population misused prescription opioids. OpiAID received an SBIR Phase I award in September 2020 from NIDA to study the biometrics of individuals receiving MOUD treatment. In April 2021, OpiAID’s CEO and COO participated in the NIH Innovation Corps (I-Corps) program to collect stakeholder feedback on leveraging clinical decision-support tools to care for patients with OUD.

Clinics and outpatient programs exist to support individuals on their path to sobriety, however, most of these programs struggle to retain patients and maintain their engagement in their recovery process. Physicians and caregivers are tasked with striking the balance of managing medication while avoiding potential withdrawal symptoms, unearthing, and addressing additional behavioral and physical health concerns, and coaching their patients to pursue behavioral health and social support efforts like individual or group therapy. These difficulties prompted OpiAID to develop a software solution to inform and support real-time clinician decision-making with actionable insights to provide an enhanced patient experience that could reduce relapses, better manage withdrawal symptoms, and ultimately improve retention.

OpiAID interviewed 77 MOUD providers, 20 patients with OUD, 7 emergency room physicians, 4 Quick Response Team member (QRT), and 6 social workers.

Major takeaways from the I-Corps interviews included the following:

1. There is a clear need for evidence-based, personalized substitution treatments for opioid dependence. While generalized MOUD guidelines exist, no standardized policy supports individualized approaches with agonist substitutes, limiting effective, tailored care. Physicians prescribing methadone or buprenorphine for MOUD should ideally tailor medication type and dosage to each patient, but such individualized considerations are often unavailable.
2. Patient monitoring is critical for effective medication induction during MOUD. However, there is no standard of care providing meaningful insight into effective patient monitoring, leaving some clinics to rely on texting and calling patients. Effective patient monitoring is essential during MOUD induction, yet no approved devices exist to guide this process. This gap forces some clinics to rely on basic methods like texting and calling patients, creating a significant barrier to providing effective monitoring for patients.
3. If the ability to remotely monitor acute opioid use and withdrawal in patients undergoing MOUD were available it would greatly enhance the safety and efficacy of OUD treatment programs. Specifically, it would allow for better management of withdrawal symptoms and support clinicians in making dosing decisions, particularly during the induction phase of MOUD treatment.
4. Identifying recent use, withdrawal, and vulnerable states (e.g., craving), is viewed as critical to clinical decision-making regarding the provision of resources and determining the level of care. For instance, measures, such as a “Just-in-Time” (JIT) alert that can notify clinicians to not administer opioids during periods of withdrawal, can prevent relapse or discontinuation of care,
5. Physicians will be motivated to use this software because of both the potential for improved outcomes and the potential for a positive impact on clinic revenue. Remote Patient Monitoring is a billable Current Procedural Terminology (CPT) code that has a national average of around $186 per patient per month. This is an emerging billing opportunity in SUD care, though its most widely utilized within cardiology and nephrology.
6. Many clinicians in the outpatient MOUD treatment setting expressed the need for timely and actionable data to support clinical decision-making.

The NIDA I-Corps SBIR Phase I funding helped demonstrate the feasibility of identifying use and withdrawal in patients undergoing MOUD for OUD through a wearable biometric device and resulted in the creation of the SaMD component of the OpiAID solution, also known as the Strength Band Platform, a software platform that remotely monitors the physiological parameters of patients receiving MOUD.

## Discussion

This study presents market research collected by four NIDA-funded companies that participated in the Spring 2021 I-Corps cohort. These data represent over 400 interviews with clinicians and other major stakeholders within the OUD community. The combined findings identify and refine barriers and opportunities in the development, commercialization and utilization of interventions for OUD and the opioid crisis.

Several barriers were consistently identified across all intervention types, therapeutics, medical device, and SaMD (Table 2). First, the management of patients with OUD lacks standardization across medical providers [25] and complicates planning for market entry and product launch. Without a standard of care, which OUD interventions a physician employs are heavily influenced by resources, geography, policy changes and the individual patient-provider relationship. While financial pressures on hospital systems may drive the adoption of new interventions, the availability of generic therapeutics and social services means new OUD interventions must be accompanied by a strong pharmacoeconomic justification for adoption. Without financial and business support from institutions like NIH I-Corps, creating this justification in a highly fragmented treatment environment is financially risky to an early-stage startup. Product teams must invest substantial early resources to understand specific care gaps prior to product validation. Alternatively, teams could attempt to design interventions that can be adapted to multiple medical environments and treatment protocols, which often increases development complexity, time to market and required capital. The challenges a fragmented landscape presents to product adoption are exemplified by other programs’ disappointing initial market penetration.

**Table 2:** Barriers to commercialization/implementation identified during the NIDA I-Corps interviews

Barrier Category	Specific Barrier
Systemic	• Presence of the X-waiver* • Limited access to insurance coverage for OUD-specific treatment • Laws and consequences of OUD use varying state-by-state
Patient Care	• Differences in OUD treatment types available based on geographic region • Lack of precision care for OUD • Lack of proper OUD patient surveillance • Need for at-home OD device data collection
Financial	• OUD patient readmissions are a major cost factor for academic medical centers • Liability and uncertainty regarding funding for OUD innovation
Social	• Stigma from providers in prescribing MOUD • Limited resources for OUD-based harm reduction services • Psychological unpreparedness toward OUD treatment from patients • Need for a social support structure to prevent OD

* The findings presented in this paper occurred before the repeal of the X-waiver

Second, underlying SDOH are dominant factors contributing to poor outcomes in OUD and will influence the potential success of newly commercialized interventions in this space [26]. Since a large portion of OUD patients lack insurance coverage, any product’s reimbursement and distribution strategy must account for this reality. Moreover, housing instability among individuals in this population makes it difficult to monitor patients and collect actionable data for clinical decisions [27, 28]. The gaps in continuity of care present an opportunity for innovative solutions that deliver meaningful clinical value and integrate into existing workflows. The success of naloxone vending machines demonstrates how harm reduction-informed interventions can effectively reach vulnerable populations.

Third, new interventions will contend with entrenched stigma and social barriers that decrease patient motivation for seeking and maintaining treatment [29, 30]. The path to recovery is not an easy choice when a patient risks compromising their personal reputation, employment status and connection with friends and family. Therefore, product design must prioritize patient privacy and social concerns, as demonstrated by Ayuda Medical’s pivot to patient-designated contacts instead of automatic 911 calls.

Lastly, federal, state, and local policies are constantly evolving and it is imperative for OUD innovators to maintain an awareness of public health trends and legal changes. A notable example is the evolution of fentanyl test strip policy - these life-saving tools were once criminalized as drug paraphernalia, but policy reforms have increasingly decriminalized their use, enabling them to serve a vital role in OD prevention. Similarly, when COVID-19 disrupted traditional treatment programs, policy adaptations enabled telemedicine and remote care options, as demonstrated by the expansion of virtual OUD treatment providers like Ophelia Health, Inc.

The worsening opioid crisis requires these barriers to OUD market entry be urgently addressed through policy changes and thoughtful product design. Seven months after the conclusion of the 2021 I-Corps course, the Centers for Disease Control and Prevention (CDC) announced that more than 100,000 people died in the U.S. from an OD between April 2020 and April 2021. This spurred several actions by the federal government between 2021 and 2023, including the repeal of the X-waiver requirement, the Office of National Drug Control Policy (ONDCP) naloxone saturation plan, and major initiatives to reduce the stigma associated with OUD and seeking treatment. Policy changes (e.g. telemedicine rules), however, have not remained as durable as the harm reduction and healthcare provider communities would have hoped. To further complicate OD mitigation efforts, Waves 3 and 4 of the opioid crisis have raised the stakes with fentanyl contaminating an increasing percentage of the illicit drug supply (heroin, methamphetamines, cocaine) [31–33]. The rise of fentanyl poisonings has outpaced all other modes of OD, leading to a need for higher doses of naloxone to be used for OD reversal [34]. Pervasive fentanyl exposure is likely to reduce the success of transitioning individuals from active use to treatment, though this detrimental impact has yet to be calculated. Given these rapid shifts in both policy and community needs, innovators must maintain ongoing stakeholder engagement to ensure their solutions remain relevant and effective.

While our research encompassed over 400 stakeholder interviews, several limitations must be acknowledged. Most importantly, while one company conducted interviews with individuals with lived experience, the collective dataset lacks robust representation from this crucial stakeholder group, which is essential for understanding ongoing recovery challenges. Additionally, I-Corps methodology relied heavily on participants’ professional networks to secure interviews, rather than a systematically curated participant pool. This approach may have introduced selection bias and failed to screen for potential conflicts of interest. The week-to-week adaptive nature of the course allowed for iterative learning; however interview questions were updated frequently, and prevented standardization that would enable pooling and rigorous statistical analysis. It is also important to note that one group of the I-Corps program did not include people with OUD.

As a direct result of the NIH I-Corps experience, the authors of this paper have launched The Substance Use Disorder Solutions Network (SUDSN) to support the early development and commercialization of evidenced-based interventions for the SUD/OUD community. This initiative seeks to expand educational and data collection resources, publish insights targeted to help healthcare professionals, policymakers and entrepreneurs working in the SUD space and build a collaborative community of innovators and entrepreneurs who deeply understand specific challenges faced by SUD communities. SUDSN ultimately aims to help bridge the gaps that currently limit the development and deployment of effective interventions.

## Conclusion

While OUD therapeutics, devices, and digital health solutions exist, their widespread adoption has been limited by significant commercialization and market entry barriers. Our findings emphasize that successful program development requires early and sustained engagement with key stakeholders across the OUD ecosystem - from patients, clinicians and policymakers to regulators, payors, manufacturers, and investors. This engagement is crucial for identifying genuine unmet needs and strategically positioning innovations for market success. The NIH I-Corps program has demonstrated its critical value by providing a structured framework that enables professionals from diverse backgrounds to contribute meaningfully to both building businesses and developing products to address the ongoing opioid crisis.

## Data Availability

No datasets were generated or analysed during the current study.

## Abbreviations

OUD: Opioid use disorder
NIDA: National institute on drug abuse
NIH: National institute on drug abuse
I-Corps: Innovation-Corps
SaMD: Software as a medical device
SDOH: Social determinants of health
MOUD: Medication for opioid use disorder
MAT: Medication-assisted treatment
SBIR: Small business innovation research
STTR: Small business technology transfer
HEAL: Helping to end addiction long-term
OAT: Opioid agonist treatment
FDA: Food and drug administration
OD: Overdose
SUD: Substance use disorder
MVP: Minimal viable product
QRT: Quick response teams
CDC: Centers for disease control and prevention
ONDCP: Office of national drug control policy
ED: Emergency department
SUDSN: The substance use disorder solutions network
